# 1,2-Bis[5-(9-ethyl-9*H*-carbazol-3-yl)-2-methyl­thio­phen-3-yl]-3,3,4,4,5,5-hexa­fluoro­cyclo­pentene

**DOI:** 10.1107/S1600536811029539

**Published:** 2011-07-30

**Authors:** Koji Kubono, Teruo Synmyouzu, Kenta Goto, Tsuyoshi Tsujioka, Keita Tani

**Affiliations:** a Division of Natural Sciences, Osaka Kyoiku University, Kashiwara, Osaka 582-8582, Japan; b Institute for Materials Chemistry and Engineering, Kyushu University, Hakozaki 6-10-1, Higashi-ku, Fukuoka 812-8581, Japan

## Abstract

The title compound, C_43_H_32_F_6_N_2_S_2_, is a new symmetrical photochromic diaryl­ethene derivative with 9-ethyl­carbazol-3-yl substituents. The mol­ecule adopts a photoactive anti­parallel conformation [Irie (2000). *Chem. Rev.* **100**, 1685–1716; Kobatake *et al.* (2002). *Chem. Commun.* pp. 2804–2805], with a dihedral angle between the mean planes of the two thio­phene rings of 56.23 (6)°. The distance between the two reactive C atoms is 3.497 (3) Å. In the crystal, two mol­ecules are associated through a pair of C—H⋯F inter­molecular hydrogen bonds, forming a centrosymmetric dimer. Dimers are linked by weak π–π inter­actions [centroid–centroid distance = 3.8872 (13) Å], forming chains along the *c* axis.

## Related literature

For a review of diaryl­ethenes, see: Irie (2000 ▶). For related structures, see: Irie *et al.* (1995 ▶, 2001 ▶); Kobatake *et al.* (2002 ▶); Takami & Irie *et al.* (2004 ▶). For a review of carbazole, see: Grigalevicius (2006 ▶). For hydrogen-bond motifs, see: Bernstein *et al.* (1995 ▶).
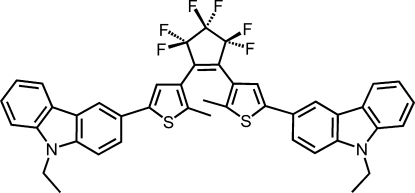


## Experimental

### 

#### Crystal data

C_43_H_32_F_6_N_2_S_2_
*M* *_r_* = 754.85
Monoclinic, 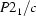

*a* = 14.6687 (7) Å
*b* = 17.0977 (8) Å
*c* = 14.0017 (7) Å
β = 95.798 (3)°
*V* = 3493.7 (3) Å^3^
*Z* = 4
Cu *K*α radiation
μ = 1.97 mm^−1^
*T* = 123 K
0.34 × 0.18 × 0.11 mm

#### Data collection

Rigaku R-AXIS RAPID diffractometer
Absorption correction: multi-scan (*ABSCOR*; Higashi, 1995 ▶) *T*_min_ = 0.687, *T*_max_ = 0.806
40777 measured reflections
6393 independent reflections
5482 reflections with *F* ^2^ > 2σ(*F* ^2^)
*R*_int_ = 0.045

#### Refinement

*R*[*F* ^2^ > 2σ(*F* ^2^)] = 0.049
*wR*(*F* ^2^) = 0.139
*S* = 1.00
6393 reflections
479 parameters
H-atom parameters constrained
Δρ_max_ = 0.90 e Å^−3^
Δρ_min_ = −0.50 e Å^−3^

### 

Data collection: *PROCESS-AUTO* (Rigaku, 2006 ▶); cell refinement: *PROCESS-AUTO*; data reduction: *CrystalStructure* (Rigaku/MSC, 2006 ▶); program(s) used to solve structure: *SIR92* (Altomare *et al.*, 1993 ▶); program(s) used to refine structure: *SHELXL97* (Sheldrick, 2008 ▶); molecular graphics: *PLATON* (Spek, 2009 ▶); software used to prepare material for publication: *CrystalStructure*.

## Supplementary Material

Crystal structure: contains datablock(s) global, I. DOI: 10.1107/S1600536811029539/bv2188sup1.cif

Structure factors: contains datablock(s) I. DOI: 10.1107/S1600536811029539/bv2188Isup2.hkl

Supplementary material file. DOI: 10.1107/S1600536811029539/bv2188Isup3.cml

Additional supplementary materials:  crystallographic information; 3D view; checkCIF report

## Figures and Tables

**Table 1:** Hydrogen-bond geometry (Å, °)

*D*—H⋯*A*	*D*—H	H⋯*A*	*D*⋯*A*	*D*—H⋯*A*
C26—H26⋯F2^i^	0.95	2.44	3.290 (2)	149 (1)

Symmetry code: (i) 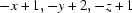.

**Fig. 1.**
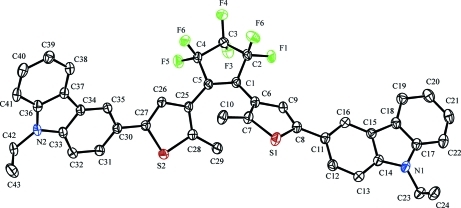
The molecular structure of (I) with the atom-labelling scheme and displacement ellipsoids are drawn at the 50% probability level. H atoms are omitted for clarity.

**Fig. 2.**
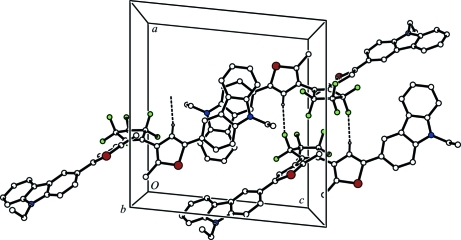
A packing diagram of (I), viewed down the b axis. The C—H···F hydrogen bonds are shown as dashed lines.

## supplementary crystallographic information

### Comment

Diarylethenes are well known photochromic compounds both in solution and in solid state (Irie, 2000), and have attracted much attention because of their potential application to optical memory, photoswitches (Irie, *et al.* 2001), and display devices (Takami & Irie, 2004). It was reported that diarylethenes can undergo a photochemical ring-closure reaction in the crystalline phase when the ring-opening forms are in the anti-parallel conformation and where the distance between two reactive C atoms is shorter than 4.2 Å (Irie, *et al.* 1995; Kobatake, *et al.* 2002). Therefore, X-ray analysis of diarylethenes will give valuable information for their photochromism in solid state. We have prepared the title compound, (I), a symmetrical diarylethene derivative containing carbazole moiety as hole transport material (Grigalevicius, 2006) to study not only its photochromism but also its electrical properties. In this paper, the molecular and crystal structure of (I) is presented.

In the molecular structure of (I), the thiophene rings are located in a photoactive anti-parallel conformation which can effectively undergo photocyclization reactions; with the dihedral angle between the mean planes of two thiophene rings, S1/C6–C9 and S2/C26–C28, of 56.23 (6) ° (Fig. 1). The dihedral angles between the thiophene rings and adjacent carbazole moieties are 23.49 (5) ° for S1/C6–C9 and N1/C11–C22, and 23.19 (5) ° for S2/C26–C28 and N2/C30–C41. The distance between two reactive C atoms in ring-closure reaction, C7···C28, is 3.497 (3) Å. This distance is shorter than 4.2 Å, suggesting that (I) can undergo the ring-closure reaction and photochromism in the crystalline phase by UV irradiation.

In the crystal structure of (I), there are intermolecular C—H···F hydrogen bonds (Fig. 2 and Table 1). Two molecules are associated through a pair of C—H···F intermolecular hydrogen bonds, forming a centrosymmetric dimer with a *R*_2_^2^(14) ring motif (Bernstein *et al.*, 1995). In the crystal, intermolecular C···C distances between carbazole moieties for C30···C36^ii^ and C34···C34^ii^ [symmetry code: (ii) 1 - *x*, 2 - *y*, 2 - *z*] are 3.657 (3) and 3.659 (3) Å, respectively. Dimers are linked by weak π–π interactions between carbazole moieties to give one-dimensional supramolecular chains propagating along the *c* axis.

### Experimental

The title compound, (I), was prepared by the treatment of 3-bromo-5-(9-ethylcarbazolyl)-2-methylthiophene with butyl lithium, then with octafluorocyclopentene. The product was recrystallized from benzene-hexane to give plate crystals, m.p. 458–460 K; ^1^H NMR (CDCl_3_, p.p.m. 400 MHz): 1.45 (t, *J* = 7.2 Hz, 6H, Et), 2.05 (s, 6H, CH_3_), 4.38 (q, *J* = 7.2 Hz, 4H, Et), 7.20–7.24 (m, 2H, carbazole), 7.32 (s, 2H, thiophene). 7.39–7.43 (m, 2H, carbazole), 7.47–7.52 (m, 2H, carbazole), 7.67 (dd, *J* = 7.8 Hz, *J'* = 1.6 Hz, 2H, carbazole), 8.11 (d, *J* = 7.8 Hz, 2H, carbazole), 8.25 (d, *J* = 1.6 Hz, 2H, carbazole); HRMS(FAB): calculated for C_43_H_32_F_6_N_2_S_2_: 754.1911, found(*M*^+^): 754.1908.

### Refinement

All H atoms bound to C atoms were placed at idealized positions and refined as a riding atoms, with C—H = 0.93–0.97Å and *U*_iso_(H) = 1.2 *U*_eq_(C) [1.5*U*_eq_(C) for methyl H atoms]. Structure was refined with unique reflections and with a cut-off sigma = 2.00.

### Crystal data

**Table:**

C_43_H_32_F_6_N_2_S_2_	*F*(000) = 1560.00
*M**_r_* = 754.85	*D*_x_ = 1.435 Mg m^−^^3^
Monoclinic, *P*2_1_/*c*	Cu *K*α radiation, λ = 1.54187 Å
Hall symbol: -P 2ybc	Cell parameters from 36134 reflections
*a* = 14.6687 (7) Å	θ = 3.0–68.3°
*b* = 17.0977 (8) Å	µ = 1.97 mm^−^^1^
*c* = 14.0017 (7) Å	*T* = 123 K
β = 95.798 (3)°	Plate, blue
*V* = 3493.7 (3) Å^3^	0.34 × 0.18 × 0.11 mm
*Z* = 4	

### Data collection

**Table:**

Rigaku R-AXIS RAPID diffractometer	5482 reflections with *F*^2^ > 2σ(*F*^2^)
Detector resolution: 5.00 pixels mm^-1^	*R*_int_ = 0.045
ω scans	θ_max_ = 68.3°
Absorption correction: multi-scan (*ABSCOR*; Higashi, 1995)	*h* = −17→17
*T*_min_ = 0.687, *T*_max_ = 0.806	*k* = −20→20
40777 measured reflections	*l* = −16→16
6393 independent reflections	

### Refinement

**Table:**

Refinement on *F*^2^	H-atom parameters constrained
*R*[*F*^2^ > 2σ(*F*^2^)] = 0.049	*w* = 1/[σ^2^(*F*_o_^2^) + (0.0796*P*)^2^ + 3.7293*P*] where *P* = (*F*_o_^2^ + 2*F*_c_^2^)/3
*wR*(*F*^2^) = 0.139	(Δ/σ)_max_ = 0.001
*S* = 1.00	Δρ_max_ = 0.90 e Å^−^^3^
6393 reflections	Δρ_min_ = −0.50 e Å^−^^3^
479 parameters	

### Special details

**Table:**

Geometry. All e.s.d.'s (except the e.s.d. in the dihedral angle between two l.s. planes) are estimated using the full covariance matrix. The cell e.s.d.'s are taken into account individually in the estimation of e.s.d.'s in distances, angles and torsion angles; correlations between e.s.d.'s in cell parameters are only used when they are defined by crystal symmetry. An approximate (isotropic) treatment of cell e.s.d.'s is used for estimating e.s.d.'s involving l.s. planes.
Refinement. Refinement was performed using all reflections. The weighted *R*-factor (*wR*) and goodness of fit (*S*) are based on *F*^2^. *R*-factor (gt) are based on *F*. The threshold expression of *F*^2^ > 2.0 σ(*F*^2^) is used only for calculating *R*-factor (gt).

### Fractional atomic coordinates and isotropic or equivalent isotropic displacement parameters (Å^2^)

**Table:**

	*x*	*y*	*z*	*U*_iso_*/*U*_eq_	
S1	0.22266 (5)	0.77807 (4)	0.34058 (5)	0.03204 (16)	
S2	0.20529 (4)	0.90769 (4)	0.72247 (4)	0.02856 (16)	
F1	0.28611 (10)	1.07557 (9)	0.27753 (10)	0.0337 (3)	
F2	0.42244 (10)	1.03335 (9)	0.32305 (11)	0.0381 (3)	
F3	0.28436 (11)	1.17289 (9)	0.41361 (11)	0.0392 (3)	
F4	0.43102 (11)	1.17032 (10)	0.40307 (11)	0.0428 (4)	
F5	0.33901 (11)	1.13896 (8)	0.58847 (10)	0.0342 (3)	
F6	0.46135 (10)	1.07923 (9)	0.55108 (10)	0.0326 (3)	
N1	−0.06405 (14)	0.78041 (12)	−0.06739 (15)	0.0268 (4)	
N2	0.50050 (14)	0.87561 (12)	1.12292 (14)	0.0252 (4)	
C1	0.30629 (15)	0.99316 (13)	0.41816 (16)	0.0217 (4)	
C2	0.34231 (16)	1.05578 (14)	0.35643 (17)	0.0243 (4)	
C3	0.35960 (17)	1.12687 (14)	0.42239 (17)	0.0263 (5)	
C4	0.37050 (16)	1.09033 (14)	0.52264 (17)	0.0243 (5)	
C5	0.31915 (15)	1.01445 (13)	0.51163 (16)	0.0214 (4)	
C6	0.26821 (16)	0.92071 (14)	0.37434 (17)	0.0236 (4)	
C7	0.28281 (17)	0.84660 (14)	0.41198 (18)	0.0281 (5)	
C8	0.17827 (16)	0.84749 (14)	0.25868 (17)	0.0260 (5)	
C9	0.21055 (16)	0.91970 (14)	0.28574 (17)	0.0241 (4)	
C10	0.34286 (19)	0.81983 (15)	0.49802 (19)	0.0335 (5)	
C11	0.11541 (16)	0.82652 (14)	0.17425 (17)	0.0249 (5)	
C12	0.06121 (16)	0.75866 (14)	0.17428 (17)	0.0268 (5)	
C13	−0.00007 (16)	0.73789 (14)	0.09707 (18)	0.0274 (5)	
C14	−0.00796 (16)	0.78690 (14)	0.01743 (18)	0.0254 (5)	
C15	0.04468 (16)	0.85586 (14)	0.01595 (17)	0.0236 (4)	
C16	0.10621 (16)	0.87512 (14)	0.09387 (17)	0.0246 (5)	
C17	−0.04857 (16)	0.84348 (14)	−0.12509 (17)	0.0262 (5)	
C18	0.01812 (16)	0.89281 (14)	−0.07567 (17)	0.0254 (5)	
C19	0.04274 (17)	0.96190 (15)	−0.11862 (18)	0.0296 (5)	
C20	0.00271 (19)	0.97990 (16)	−0.20982 (19)	0.0341 (5)	
C21	−0.06116 (18)	0.92965 (16)	−0.25817 (19)	0.0339 (6)	
C22	−0.08871 (18)	0.86168 (16)	−0.21681 (18)	0.0321 (5)	
C23	−0.13082 (18)	0.71836 (16)	−0.0910 (2)	0.0331 (5)	
C24	−0.0899 (2)	0.64855 (16)	−0.1365 (2)	0.0391 (6)	
C25	0.29322 (16)	0.97565 (13)	0.59806 (16)	0.0217 (4)	
C26	0.35396 (16)	0.96925 (13)	0.68333 (16)	0.0233 (4)	
C27	0.31788 (16)	0.93281 (13)	0.75725 (17)	0.0229 (4)	
C28	0.20781 (16)	0.94502 (14)	0.60858 (17)	0.0261 (5)	
C29	0.12464 (17)	0.94189 (17)	0.53832 (18)	0.0318 (5)	
C30	0.36117 (16)	0.91630 (13)	0.85442 (17)	0.0234 (4)	
C31	0.30891 (17)	0.90623 (14)	0.93247 (17)	0.0255 (5)	
C32	0.34847 (17)	0.89227 (14)	1.02435 (17)	0.0254 (5)	
C33	0.44370 (17)	0.88832 (13)	1.03932 (16)	0.0232 (4)	
C34	0.49780 (16)	0.89756 (13)	0.96205 (16)	0.0228 (4)	
C35	0.45622 (17)	0.91139 (13)	0.87016 (17)	0.0237 (4)	
C36	0.59047 (17)	0.87628 (13)	1.10144 (17)	0.0254 (5)	
C37	0.59257 (16)	0.88950 (13)	1.00191 (17)	0.0238 (4)	
C38	0.67583 (17)	0.89345 (15)	0.96312 (18)	0.0285 (5)	
C39	0.75581 (17)	0.88401 (15)	1.02312 (19)	0.0315 (5)	
C40	0.75293 (18)	0.87091 (16)	1.12114 (19)	0.0341 (5)	
C41	0.67171 (18)	0.86625 (15)	1.16179 (18)	0.0305 (5)	
C42	0.47018 (19)	0.86212 (15)	1.21760 (17)	0.0290 (5)	
C43	0.4644 (2)	0.77700 (17)	1.2437 (2)	0.0450 (7)	
H9	0.1960	0.9655	0.2489	0.029*	
H10A	0.3375	0.7630	0.5046	0.040*	
H10B	0.3240	0.8454	0.5555	0.040*	
H10C	0.4066	0.8335	0.4906	0.040*	
H12	0.0670	0.7259	0.2294	0.032*	
H13	−0.0357	0.6916	0.0983	0.033*	
H16	0.1421	0.9213	0.0926	0.030*	
H19	0.0863	0.9962	−0.0860	0.036*	
H20	0.0189	1.0270	−0.2398	0.041*	
H21	−0.0863	0.9427	−0.3214	0.041*	
H22	−0.1334	0.8285	−0.2496	0.039*	
H23A	−0.1817	0.7393	−0.1356	0.040*	
H23B	−0.1567	0.7015	−0.0317	0.040*	
H24A	−0.1374	0.6088	−0.1511	0.047*	
H24B	−0.0405	0.6268	−0.0921	0.047*	
H24C	−0.0653	0.6647	−0.1960	0.047*	
H26	0.4149	0.9888	0.6884	0.028*	
H29A	0.0748	0.9162	0.5679	0.038*	
H29B	0.1383	0.9123	0.4815	0.038*	
H29C	0.1061	0.9952	0.5193	0.038*	
H31	0.2440	0.9092	0.9214	0.031*	
H32	0.3119	0.8855	1.0760	0.030*	
H35	0.4925	0.9175	0.8182	0.028*	
H38	0.6778	0.9025	0.8964	0.034*	
H39	0.8133	0.8865	0.9974	0.038*	
H40	0.8089	0.8650	1.1610	0.041*	
H41	0.6707	0.8566	1.2285	0.037*	
H42A	0.4091	0.8862	1.2198	0.035*	
H42B	0.5132	0.8886	1.2663	0.035*	
H43A	0.4440	0.7722	1.3079	0.054*	
H43B	0.5249	0.7528	1.2431	0.054*	
H43C	0.4206	0.7506	1.1970	0.054*	

### Atomic displacement parameters (Å^2^)

**Table:**

	*U*^11^	*U*^22^	*U*^33^	*U*^12^	*U*^13^	*U*^23^
S1	0.0406 (3)	0.0251 (3)	0.0276 (3)	−0.0005 (2)	−0.0101 (2)	−0.0004 (2)
S2	0.0251 (3)	0.0382 (3)	0.0217 (3)	−0.0050 (2)	−0.0011 (2)	0.0071 (2)
F1	0.0398 (8)	0.0397 (8)	0.0191 (7)	−0.0037 (6)	−0.0086 (6)	0.0084 (6)
F2	0.0342 (8)	0.0433 (8)	0.0394 (9)	0.0045 (6)	0.0168 (7)	0.0049 (6)
F3	0.0533 (9)	0.0322 (8)	0.0310 (8)	0.0156 (7)	−0.0006 (7)	0.0054 (6)
F4	0.0499 (9)	0.0477 (9)	0.0298 (8)	−0.0243 (7)	−0.0007 (7)	0.0087 (6)
F5	0.0527 (9)	0.0269 (7)	0.0232 (7)	−0.0022 (6)	0.0045 (6)	−0.0030 (5)
F6	0.0269 (7)	0.0406 (8)	0.0281 (7)	−0.0080 (6)	−0.0077 (6)	0.0069 (6)
N1	0.0246 (10)	0.0311 (10)	0.0234 (10)	−0.0023 (8)	−0.0044 (8)	−0.0039 (8)
N2	0.0310 (10)	0.0295 (10)	0.0144 (9)	0.0024 (8)	−0.0012 (8)	0.0004 (7)
C1	0.0197 (10)	0.0258 (11)	0.0189 (11)	0.0023 (8)	−0.0020 (8)	0.0019 (9)
C2	0.0209 (11)	0.0331 (12)	0.0183 (11)	0.0031 (9)	−0.0010 (9)	0.0026 (9)
C3	0.0283 (12)	0.0272 (12)	0.0229 (12)	−0.0027 (9)	−0.0004 (9)	0.0062 (9)
C4	0.0251 (11)	0.0289 (12)	0.0177 (11)	−0.0002 (9)	−0.0027 (9)	−0.0001 (9)
C5	0.0196 (10)	0.0249 (11)	0.0189 (11)	0.0020 (8)	−0.0019 (8)	0.0022 (8)
C6	0.0245 (11)	0.0272 (11)	0.0185 (11)	0.0015 (9)	−0.0013 (9)	0.0004 (9)
C7	0.0322 (13)	0.0266 (12)	0.0239 (12)	0.0009 (9)	−0.0054 (10)	−0.0012 (9)
C8	0.0258 (11)	0.0295 (12)	0.0218 (12)	0.0016 (9)	−0.0026 (9)	0.0010 (9)
C9	0.0269 (12)	0.0267 (11)	0.0180 (11)	0.0015 (9)	−0.0011 (9)	0.0003 (9)
C10	0.0382 (14)	0.0282 (12)	0.0312 (14)	0.0046 (10)	−0.0108 (11)	−0.0018 (10)
C11	0.0234 (11)	0.0282 (12)	0.0224 (12)	0.0017 (9)	−0.0014 (9)	−0.0013 (9)
C12	0.0291 (12)	0.0281 (12)	0.0225 (12)	0.0006 (9)	−0.0002 (10)	0.0017 (9)
C13	0.0270 (12)	0.0280 (12)	0.0270 (13)	−0.0037 (9)	0.0011 (10)	−0.0016 (9)
C14	0.0228 (11)	0.0280 (12)	0.0251 (12)	0.0006 (9)	0.0007 (9)	−0.0053 (9)
C15	0.0229 (11)	0.0260 (11)	0.0221 (12)	0.0001 (8)	0.0031 (9)	−0.0018 (9)
C16	0.0237 (11)	0.0248 (11)	0.0250 (12)	−0.0013 (9)	0.0002 (9)	−0.0017 (9)
C17	0.0251 (11)	0.0294 (12)	0.0240 (12)	0.0044 (9)	0.0010 (9)	−0.0041 (9)
C18	0.0249 (11)	0.0293 (12)	0.0219 (12)	0.0037 (9)	0.0026 (9)	−0.0016 (9)
C19	0.0300 (12)	0.0308 (12)	0.0282 (13)	0.0006 (10)	0.0032 (10)	−0.0015 (10)
C20	0.0386 (14)	0.0347 (13)	0.0290 (14)	0.0076 (11)	0.0040 (11)	0.0061 (10)
C21	0.0364 (14)	0.0424 (14)	0.0221 (13)	0.0139 (11)	−0.0007 (11)	0.0024 (11)
C22	0.0291 (12)	0.0405 (14)	0.0255 (13)	0.0076 (10)	−0.0032 (10)	−0.0055 (11)
C23	0.0278 (12)	0.0384 (14)	0.0317 (14)	−0.0070 (10)	−0.0043 (10)	−0.0019 (11)
C24	0.0435 (16)	0.0335 (14)	0.0381 (16)	−0.0071 (11)	−0.0072 (12)	−0.0054 (11)
C25	0.0240 (11)	0.0217 (10)	0.0184 (11)	0.0009 (8)	−0.0028 (9)	0.0007 (8)
C26	0.0247 (11)	0.0261 (11)	0.0182 (11)	−0.0027 (9)	−0.0024 (9)	0.0007 (9)
C27	0.0240 (11)	0.0229 (11)	0.0214 (11)	−0.0013 (8)	−0.0000 (9)	0.0011 (9)
C28	0.0261 (12)	0.0300 (12)	0.0212 (12)	−0.0021 (9)	−0.0017 (9)	0.0025 (9)
C29	0.0244 (12)	0.0454 (15)	0.0245 (13)	−0.0013 (10)	−0.0038 (10)	0.0073 (11)
C30	0.0273 (12)	0.0228 (11)	0.0192 (11)	−0.0004 (9)	−0.0014 (9)	0.0011 (8)
C31	0.0255 (12)	0.0268 (12)	0.0238 (12)	0.0008 (9)	0.0006 (9)	0.0018 (9)
C32	0.0292 (12)	0.0271 (11)	0.0202 (12)	0.0004 (9)	0.0041 (9)	0.0028 (9)
C33	0.0310 (12)	0.0216 (11)	0.0167 (11)	−0.0001 (9)	0.0010 (9)	−0.0002 (8)
C34	0.0275 (12)	0.0225 (10)	0.0179 (11)	0.0001 (8)	−0.0009 (9)	0.0004 (8)
C35	0.0279 (12)	0.0251 (11)	0.0181 (11)	−0.0015 (9)	0.0017 (9)	0.0006 (9)
C36	0.0317 (12)	0.0229 (11)	0.0210 (12)	0.0023 (9)	−0.0004 (10)	−0.0017 (9)
C37	0.0273 (12)	0.0233 (11)	0.0199 (11)	0.0014 (9)	−0.0025 (9)	−0.0012 (9)
C38	0.0322 (13)	0.0329 (12)	0.0198 (12)	0.0025 (10)	−0.0008 (10)	−0.0008 (10)
C39	0.0264 (12)	0.0365 (13)	0.0309 (14)	0.0039 (10)	−0.0008 (10)	−0.0051 (10)
C40	0.0324 (13)	0.0362 (14)	0.0311 (14)	0.0075 (10)	−0.0089 (11)	−0.0053 (11)
C41	0.0361 (13)	0.0337 (13)	0.0195 (12)	0.0049 (10)	−0.0075 (10)	−0.0026 (10)
C42	0.0388 (14)	0.0341 (13)	0.0141 (11)	0.0055 (10)	0.0023 (10)	−0.0026 (9)
C43	0.069 (2)	0.0389 (15)	0.0292 (14)	−0.0049 (14)	0.0183 (14)	0.0001 (11)

### Geometric parameters (Å, °)

**Table:**

S1—C7	1.724 (2)	C30—C31	1.407 (3)
S1—C8	1.731 (2)	C30—C35	1.392 (3)
S2—C27	1.728 (2)	C31—C32	1.378 (3)
S2—C28	1.722 (2)	C32—C33	1.393 (3)
F1—C2	1.353 (2)	C33—C34	1.414 (3)
F2—C2	1.363 (2)	C34—C35	1.387 (3)
F3—C3	1.351 (2)	C34—C37	1.451 (3)
F4—C3	1.334 (3)	C36—C37	1.415 (3)
F5—C4	1.357 (2)	C36—C41	1.400 (3)
F6—C4	1.365 (2)	C37—C38	1.388 (3)
N1—C14	1.379 (3)	C38—C39	1.382 (3)
N1—C17	1.380 (3)	C39—C40	1.395 (3)
N1—C23	1.459 (3)	C40—C41	1.374 (3)
N2—C33	1.384 (2)	C42—C43	1.505 (3)
N2—C36	1.383 (3)	C9—H9	0.950
N2—C42	1.459 (3)	C10—H10A	0.980
C1—C2	1.505 (3)	C10—H10B	0.980
C1—C5	1.353 (3)	C10—H10C	0.980
C1—C6	1.468 (3)	C12—H12	0.950
C2—C3	1.532 (3)	C13—H13	0.950
C3—C4	1.530 (3)	C16—H16	0.950
C4—C5	1.500 (3)	C19—H19	0.950
C5—C25	1.464 (3)	C20—H20	0.950
C6—C7	1.381 (3)	C21—H21	0.950
C6—C9	1.429 (3)	C22—H22	0.950
C7—C10	1.491 (3)	C23—H23A	0.990
C8—C9	1.362 (3)	C23—H23B	0.990
C8—C11	1.468 (3)	C24—H24A	0.980
C11—C12	1.407 (3)	C24—H24B	0.980
C11—C16	1.395 (3)	C24—H24C	0.980
C12—C13	1.381 (3)	C26—H26	0.950
C13—C14	1.390 (3)	C29—H29A	0.980
C14—C15	1.411 (3)	C29—H29B	0.980
C15—C16	1.384 (3)	C29—H29C	0.980
C15—C18	1.448 (3)	C31—H31	0.950
C17—C18	1.418 (3)	C32—H32	0.950
C17—C22	1.393 (3)	C35—H35	0.950
C18—C19	1.390 (3)	C38—H38	0.950
C19—C20	1.385 (3)	C39—H39	0.950
C20—C21	1.395 (3)	C40—H40	0.950
C21—C22	1.377 (3)	C41—H41	0.950
C23—C24	1.506 (3)	C42—H42A	0.990
C25—C26	1.420 (3)	C42—H42B	0.990
C25—C28	1.379 (3)	C43—H43A	0.980
C26—C27	1.360 (3)	C43—H43B	0.980
C27—C30	1.470 (3)	C43—H43C	0.980
C28—C29	1.489 (3)		
			
C7—S1—C8	93.12 (11)	C32—C33—C34	120.7 (2)
C27—S2—C28	93.41 (11)	C33—C34—C35	120.0 (2)
C14—N1—C17	108.79 (19)	C33—C34—C37	106.56 (19)
C14—N1—C23	125.6 (2)	C35—C34—C37	133.4 (2)
C17—N1—C23	125.6 (2)	C30—C35—C34	119.8 (2)
C33—N2—C36	108.73 (19)	N2—C36—C37	109.37 (19)
C33—N2—C42	125.5 (2)	N2—C36—C41	129.8 (2)
C36—N2—C42	125.75 (19)	C37—C36—C41	120.8 (2)
C2—C1—C5	109.96 (19)	C34—C37—C36	106.2 (2)
C2—C1—C6	120.08 (19)	C34—C37—C38	133.8 (2)
C5—C1—C6	129.9 (2)	C36—C37—C38	120.1 (2)
F1—C2—F2	105.41 (18)	C37—C38—C39	118.9 (2)
F1—C2—C1	115.25 (18)	C38—C39—C40	120.6 (2)
F1—C2—C3	110.11 (18)	C39—C40—C41	122.0 (2)
F2—C2—C1	111.20 (18)	C36—C41—C40	117.6 (2)
F2—C2—C3	109.51 (18)	N2—C42—C43	113.8 (2)
C1—C2—C3	105.34 (19)	C6—C9—H9	122.9
F3—C3—F4	107.96 (19)	C8—C9—H9	122.9
F3—C3—C2	108.86 (18)	C7—C10—H10A	109.5
F3—C3—C4	109.3 (2)	C7—C10—H10B	109.5
F4—C3—C2	113.9 (2)	C7—C10—H10C	109.5
F4—C3—C4	113.70 (19)	H10A—C10—H10B	109.5
C2—C3—C4	102.98 (18)	H10A—C10—H10C	109.5
F5—C4—F6	106.05 (17)	H10B—C10—H10C	109.5
F5—C4—C3	111.22 (18)	C11—C12—H12	118.8
F5—C4—C5	113.45 (19)	C13—C12—H12	118.8
F6—C4—C3	109.44 (19)	C12—C13—H13	121.1
F6—C4—C5	112.02 (18)	C14—C13—H13	121.0
C3—C4—C5	104.71 (18)	C11—C16—H16	120.1
C1—C5—C4	110.6 (2)	C15—C16—H16	120.1
C1—C5—C25	130.9 (2)	C18—C19—H19	120.6
C4—C5—C25	118.47 (19)	C20—C19—H19	120.5
C1—C6—C7	125.3 (2)	C19—C20—H20	119.6
C1—C6—C9	122.7 (2)	C21—C20—H20	119.6
C7—C6—C9	112.1 (2)	C20—C21—H21	119.1
S1—C7—C6	110.60 (17)	C22—C21—H21	119.1
S1—C7—C10	119.07 (17)	C17—C22—H22	121.3
C6—C7—C10	130.3 (2)	C21—C22—H22	121.3
S1—C8—C9	109.89 (17)	N1—C23—H23A	109.1
S1—C8—C11	121.97 (17)	N1—C23—H23B	109.1
C9—C8—C11	128.1 (2)	C24—C23—H23A	109.1
C6—C9—C8	114.3 (2)	C24—C23—H23B	109.1
C8—C11—C12	120.7 (2)	H23A—C23—H23B	107.8
C8—C11—C16	120.4 (2)	C23—C24—H24A	109.5
C12—C11—C16	118.9 (2)	C23—C24—H24B	109.5
C11—C12—C13	122.4 (2)	C23—C24—H24C	109.5
C12—C13—C14	117.9 (2)	H24A—C24—H24B	109.5
N1—C14—C13	129.5 (2)	H24A—C24—H24C	109.5
N1—C14—C15	109.5 (2)	H24B—C24—H24C	109.5
C13—C14—C15	120.9 (2)	C25—C26—H26	122.7
C14—C15—C16	120.1 (2)	C27—C26—H26	122.7
C14—C15—C18	106.20 (19)	C28—C29—H29A	109.5
C16—C15—C18	133.7 (2)	C28—C29—H29B	109.5
C11—C16—C15	119.8 (2)	C28—C29—H29C	109.5
N1—C17—C18	108.99 (19)	H29A—C29—H29B	109.5
N1—C17—C22	129.4 (2)	H29A—C29—H29C	109.5
C18—C17—C22	121.6 (2)	H29B—C29—H29C	109.5
C15—C18—C17	106.5 (2)	C30—C31—H31	118.8
C15—C18—C19	134.1 (2)	C32—C31—H31	118.8
C17—C18—C19	119.4 (2)	C31—C32—H32	121.0
C18—C19—C20	118.9 (2)	C33—C32—H32	121.0
C19—C20—C21	120.8 (2)	C30—C35—H35	120.1
C20—C21—C22	121.8 (2)	C34—C35—H35	120.1
C17—C22—C21	117.5 (2)	C37—C38—H38	120.6
N1—C23—C24	112.7 (2)	C39—C38—H38	120.6
C5—C25—C26	122.5 (2)	C38—C39—H39	119.7
C5—C25—C28	125.1 (2)	C40—C39—H39	119.7
C26—C25—C28	112.4 (2)	C39—C40—H40	119.0
C25—C26—C27	114.6 (2)	C41—C40—H40	119.0
S2—C27—C26	109.49 (16)	C36—C41—H41	121.2
S2—C27—C30	121.63 (18)	C40—C41—H41	121.2
C26—C27—C30	128.9 (2)	N2—C42—H42A	108.8
S2—C28—C25	110.14 (16)	N2—C42—H42B	108.8
S2—C28—C29	120.39 (18)	C43—C42—H42A	108.8
C25—C28—C29	129.5 (2)	C43—C42—H42B	108.8
C27—C30—C31	121.6 (2)	H42A—C42—H42B	107.7
C27—C30—C35	119.4 (2)	C42—C43—H43A	109.5
C31—C30—C35	119.0 (2)	C42—C43—H43B	109.5
C30—C31—C32	122.4 (2)	C42—C43—H43C	109.5
C31—C32—C33	118.1 (2)	H43A—C43—H43B	109.5
N2—C33—C32	130.1 (2)	H43A—C43—H43C	109.5
N2—C33—C34	109.2 (2)	H43B—C43—H43C	109.5
			
C7—S1—C8—C9	1.0 (2)	C9—C6—C7—C10	175.1 (2)
C7—S1—C8—C11	−179.0 (2)	S1—C8—C9—C6	−2.4 (2)
C8—S1—C7—C6	0.7 (2)	S1—C8—C11—C12	25.0 (3)
C8—S1—C7—C10	−176.9 (2)	S1—C8—C11—C16	−157.19 (19)
C27—S2—C28—C25	0.17 (19)	C9—C8—C11—C12	−155.0 (2)
C27—S2—C28—C29	−179.2 (2)	C9—C8—C11—C16	22.8 (3)
C28—S2—C27—C26	0.76 (18)	C11—C8—C9—C6	177.6 (2)
C28—S2—C27—C30	179.51 (19)	C8—C11—C12—C13	178.6 (2)
C14—N1—C17—C18	−1.0 (2)	C8—C11—C16—C15	−178.0 (2)
C14—N1—C17—C22	179.7 (2)	C12—C11—C16—C15	−0.2 (3)
C17—N1—C14—C13	179.4 (2)	C16—C11—C12—C13	0.8 (3)
C17—N1—C14—C15	0.6 (2)	C11—C12—C13—C14	−0.5 (3)
C14—N1—C23—C24	−88.1 (3)	C12—C13—C14—N1	−179.0 (2)
C23—N1—C14—C13	1.2 (4)	C12—C13—C14—C15	−0.3 (3)
C23—N1—C14—C15	−177.6 (2)	N1—C14—C15—C16	179.8 (2)
C17—N1—C23—C24	93.9 (2)	N1—C14—C15—C18	0.0 (2)
C23—N1—C17—C18	177.2 (2)	C13—C14—C15—C16	0.9 (3)
C23—N1—C17—C22	−2.0 (4)	C13—C14—C15—C18	−178.9 (2)
C33—N2—C36—C37	−0.1 (2)	C14—C15—C16—C11	−0.6 (3)
C33—N2—C36—C41	179.9 (2)	C14—C15—C18—C17	−0.7 (2)
C36—N2—C33—C32	179.6 (2)	C14—C15—C18—C19	178.0 (2)
C36—N2—C33—C34	−0.1 (2)	C16—C15—C18—C17	179.6 (2)
C33—N2—C42—C43	96.1 (2)	C16—C15—C18—C19	−1.7 (5)
C42—N2—C33—C32	0.8 (3)	C18—C15—C16—C11	179.1 (2)
C42—N2—C33—C34	−178.9 (2)	N1—C17—C18—C15	1.0 (2)
C36—N2—C42—C43	−82.5 (3)	N1—C17—C18—C19	−177.9 (2)
C42—N2—C36—C37	178.7 (2)	N1—C17—C22—C21	179.2 (2)
C42—N2—C36—C41	−1.3 (3)	C18—C17—C22—C21	0.1 (2)
C2—C1—C5—C4	−3.9 (2)	C22—C17—C18—C15	−179.6 (2)
C2—C1—C5—C25	177.4 (2)	C22—C17—C18—C19	1.5 (3)
C5—C1—C2—F1	−134.0 (2)	C15—C18—C19—C20	−179.9 (2)
C5—C1—C2—F2	106.1 (2)	C17—C18—C19—C20	−1.4 (3)
C5—C1—C2—C3	−12.4 (2)	C18—C19—C20—C21	−0.1 (3)
C2—C1—C6—C7	138.9 (2)	C19—C20—C21—C22	1.7 (4)
C2—C1—C6—C9	−41.0 (3)	C20—C21—C22—C17	−1.6 (4)
C6—C1—C2—F1	48.5 (2)	C5—C25—C26—C27	178.7 (2)
C6—C1—C2—F2	−71.4 (2)	C5—C25—C28—S2	−177.96 (18)
C6—C1—C2—C3	170.05 (19)	C5—C25—C28—C29	1.4 (4)
C5—C1—C6—C7	−38.0 (4)	C26—C25—C28—S2	−1.0 (2)
C5—C1—C6—C9	142.0 (2)	C26—C25—C28—C29	178.3 (2)
C6—C1—C5—C4	173.3 (2)	C28—C25—C26—C27	1.7 (2)
C6—C1—C5—C25	−5.4 (4)	C25—C26—C27—S2	−1.5 (2)
F1—C2—C3—F3	31.7 (2)	C25—C26—C27—C30	179.9 (2)
F1—C2—C3—F4	−88.8 (2)	S2—C27—C30—C31	−23.2 (3)
F1—C2—C3—C4	147.60 (19)	S2—C27—C30—C35	157.56 (18)
F2—C2—C3—F3	147.18 (18)	C26—C27—C30—C31	155.3 (2)
F2—C2—C3—F4	26.7 (2)	C26—C27—C30—C35	−23.9 (3)
F2—C2—C3—C4	−96.9 (2)	C27—C30—C31—C32	−178.7 (2)
C1—C2—C3—F3	−93.1 (2)	C27—C30—C35—C34	178.6 (2)
C1—C2—C3—F4	146.34 (19)	C31—C30—C35—C34	−0.7 (3)
C1—C2—C3—C4	22.7 (2)	C35—C30—C31—C32	0.5 (3)
F3—C3—C4—F5	−32.1 (2)	C30—C31—C32—C33	0.1 (2)
F3—C3—C4—F6	−148.90 (18)	C31—C32—C33—N2	179.6 (2)
F3—C3—C4—C5	90.8 (2)	C31—C32—C33—C34	−0.7 (3)
F4—C3—C4—F5	88.6 (2)	N2—C33—C34—C35	−179.7 (2)
F4—C3—C4—F6	−28.2 (2)	N2—C33—C34—C37	0.3 (2)
F4—C3—C4—C5	−148.48 (19)	C32—C33—C34—C35	0.5 (3)
C2—C3—C4—F5	−147.66 (19)	C32—C33—C34—C37	−179.5 (2)
C2—C3—C4—F6	95.5 (2)	C33—C34—C35—C30	0.1 (2)
C2—C3—C4—C5	−24.8 (2)	C33—C34—C37—C36	−0.4 (2)
F5—C4—C5—C1	140.05 (19)	C33—C34—C37—C38	−179.2 (2)
F5—C4—C5—C25	−41.0 (2)	C35—C34—C37—C36	179.6 (2)
F6—C4—C5—C1	−99.9 (2)	C35—C34—C37—C38	0.8 (4)
F6—C4—C5—C25	79.0 (2)	C37—C34—C35—C30	−179.9 (2)
C3—C4—C5—C1	18.6 (2)	N2—C36—C37—C34	0.3 (2)
C3—C4—C5—C25	−162.5 (2)	N2—C36—C37—C38	179.3 (2)
C1—C5—C25—C26	135.6 (2)	N2—C36—C41—C40	−179.0 (2)
C1—C5—C25—C28	−47.8 (3)	C37—C36—C41—C40	1.0 (3)
C4—C5—C25—C26	−43.1 (3)	C41—C36—C37—C34	−179.7 (2)
C4—C5—C25—C28	133.5 (2)	C41—C36—C37—C38	−0.7 (3)
C1—C6—C7—S1	177.90 (19)	C34—C37—C38—C39	179.0 (2)
C1—C6—C7—C10	−4.9 (4)	C36—C37—C38—C39	0.2 (3)
C1—C6—C9—C8	−177.0 (2)	C37—C38—C39—C40	−0.2 (3)
C7—C6—C9—C8	3.0 (3)	C38—C39—C40—C41	0.5 (4)
C9—C6—C7—S1	−2.1 (2)	C39—C40—C41—C36	−0.9 (3)

### Hydrogen-bond geometry (Å, °)

**Table:**

*D*—H···*A*	*D*—H	H···*A*	*D*···*A*	*D*—H···*A*
C26—H26···F2^i^	0.95	2.44	3.290 (2)	149.(1)

Symmetry codes: (i) −*x*+1, −*y*+2, −*z*+1.
